# Osteopathic Medical Student-Led Pipeline Intervention: Enhancing High School Students’ Knowledge of Postsecondary Education and Healthcare Careers

**DOI:** 10.51894/001c.164404

**Published:** 2026-08-01

**Authors:** Javier A. Tobar, Joseph Noeske, Joshua Bertman, Aubrey Everett, Carolina Restini

**Affiliations:** 1 College of Osteopathic Medicine, Michigan State University

## 49

### INTRODUCTION

An osteopathic medical student (OMS)–led program was developed to increase awareness among high school students about the diversity of healthcare careers. Adolescents often associate healthcare careers primarily with physicians and may view the extensive educational pathway to becoming a physician as a barrier. Consequently, other healthcare professions are frequently under recognized.

### OBJECTIVES

In this study, we evaluated high school students’ baseline knowledge of health careers and assessed whether an educational intervention improved their understanding of healthcare careers such as physician assistant (PA), medical doctor (MD), Doctor of Osteopathic Medicine (DO), and nursing.

###  METHODS

This cross-sectional analytical study (IRB #00007491) included high school students from East Lansing and greater Grand Rapids enrolled in the program “Future DOcs”, at MSUCOM. Participants completed an anonymous 20-question survey before and after a standardized presentation led by OMS. Demographic data was assessed, as well as general knowledge about health care careers including osteopathic vs. allopathic medicine. Data were analyzed using descriptive statistics and chi-square tests (95% CI). 

### RESULTS

Thirty-eight students completed paired pre/post presentation surveys across two Future DOcs cohorts (12/5/2025 and 10/11/2025). Students’ baseline knowledge of the education requirements for MD, DO, and PA careers was similar. However, details about nursing educational requirements after high school had the lowest baseline accuracy (Nursing 24%, *p* < 0.0001 vs MD, DO, and PA 75%, *p* > 0.05 ) and showed a significant improvement in correct responses after the presentation (81%, *p* < 0.0001).

### DISCUSSION

This study demonstrates that targeted educational interventions within pipeline programs effectively correct misconceptions regarding healthcare training timelines. Despite nursing being a widely known healthcare profession, students demonstrated limited understanding of its required educational pathway. The presentations led by OMS significantly improved knowledge of the minimum education required for nursing. Although other careers did not significantly improve, overall healthcare career knowledge increased. This highlights the importance of early exposure to diverse medical careers. Providing high school students with accurate information about degree requirements specifically that not all healthcare roles require decade long training may increase interest in essential roles such as nursing and strengthen the future healthcare workforce.

